# Cyclin D1 sensitizes myeloma cells to endoplasmic reticulum stress-mediated apoptosis by activating the unfolded protein response pathway

**DOI:** 10.1186/s12885-015-1240-y

**Published:** 2015-04-11

**Authors:** Sophie Bustany, Julie Cahu, Philippe Guardiola, Brigitte Sola

**Affiliations:** 1 Normandie Univ, UNICAEN, EA4652, Caen, France; 2 Plateforme SNP, Transcriptome & Epigénomique, CHU, Angers, France

**Keywords:** Plasma cells, Anti-myeloma drugs, Unfolded protein response, Endoplasmic reticulum stress, Caspase-dependent apoptosis

## Abstract

**Background:**

Cyclin D1 and its kinase partners control cell cycle progression. Cyclin D1 is frequently deregulated in various cancers, including malignant hemopathies, and tumor cells display uncontrolled cell proliferation. Cyclin D1 is not expressed in the B-cell lineage but is found in multiple myeloma (MM) cells in almost 50% of patients with this condition. Paradoxically, cyclin D1 expression is associated with a good prognosis and longer overall survival in MM patients.

**Methods:**

We used two independent MM cell lines (RPMI 8226 and LP1) to generate several clones stably expressing either the green fluorescent protein (GFP) or a GFP-cyclin D1 fusion protein, and we analyzed the properties acquired following cyclin D1 expression.

**Results:**

Whole-genome expression analysis in the cell clones indicated that cyclin D1 profoundly modified several cellular functions, including the regulation of apoptotic cell death. We studied the apoptotic response of GFP- and GFP-cyclin D1-expressing clones to bortezomib-treatment. We found that the apoptotic response occurred faster and was of a greater amplitude in cyclin D1-expressing cells. Cyclin D1 expression enhanced the caspase-dependent apoptosis mediated by the intrinsic mitochondrial pathway. More importantly, cyclin D1 also activated the unfolded protein response (UPR) and induced endoplasmic reticulum (ER) stress-mediated apoptosis.

**Conclusion:**

The ER is well known to be a crucial regulator of plasma cell death and it plays the same role in their malignant counterparts, myeloma cells. This role involves activation of the UPR controlled at least in part by cyclin D1.

**Electronic supplementary material:**

The online version of this article (doi:10.1186/s12885-015-1240-y) contains supplementary material, which is available to authorized users.

## Background

The *CCND1* gene encoding cyclin D1 is the second most frequently amplified locus in solid cancers [1]. Moreover, cyclin D1 is overexpressed in human cancers, including malignant hemopathies, after genetic alterations, such as chromosomal translocation, but also in the absence of any detectable genetic alteration [2]. Tumor cells with high cyclin D1 levels have higher proliferation rate and lower nutrient requirements that tumor cells that do not express cyclin D1. This is consistent with the well known function of cyclin D1 in cell cycle regulation through cyclin-dependent kinase 4/6 activation [3]. However, the role of cyclin D1 in oncogenesis might not be limited to the increase in proliferation. Indeed, depending on its subcellular distribution (nuclear, cytoplasmic, at the outer mitochondrial membrane) and partners (transcription factors, chromatin-modifying enzymes, cytosolic proteins), cyclin D1 can regulate transcriptional regulation [4], DNA damage response [5,6], centrosome duplication [7], chromosomal instability [8], senescence [9], mitochondrial function [10] and migration [11-13]. All these processes, if left uncontrolled, can initiate or/and maintain transformation processes.

In 15% of patients with multiple myeloma (MM), a hematological disease characterized by the accumulation of malignant plasma cells in the bone marrow, cyclin D1 is aberrantly expressed as a result of the t(11;14)(q13;q32) translocation in [14]. Moreover, biallelic cyclin D1 expression is detected in 40% of MM cases, most displaying hyperdiploidy [15]. Consistent with its role in cell cycle regulation, cyclin D1 has been shown to regulate MM cell proliferation [16]. Paradoxically, MM patients with cyclin D1-expressing tumor cells have a good prognosis and a longer overall survival [17]. The possibility of additional functions for cyclin D1 in MM cells is key issue that has been little addressed. We investigated this possibility, by generating stable MM cell line-derived clones expressing a cyclin D1-green fluorescent protein (GFP) fusion protein (D1-GFP) or GFP alone. We used arrays to investigate gene expression in D1-GFP- and GFP-expressing cells. We found that the presence of cyclin D1 altered the expression of genes involved in metabolism, membrane trafficking, adhesion/migration, cell proliferation, inflammation, and cell death/apoptosis. We also found that cyclin D1 expression was sufficient to sensitize MM cells to the induction of apoptosis by bortezomib. This greater sensitivity of cyclin D1-expressing cells was mediated by the activation of the unfolded protein response (UPR) pathway and endoplasmic reticulum (ER)-stress signaling, triggering apoptosis. Our data reveal a novel molecular mechanism by which cyclin D1 expression directly targets the UPR, enhancing the response to bortezomib in MM tumor cells, as highlighting by clinical observations.

## Methods

### Chemicals

Bortezomib and Z-LEVD [Z-LE(OMe)VD(OMe)-FMK], a caspase 4 inhibitor, were purchased from Euromedex. Q-VD-OPh [quinoyl-valyl-O-methylaspartyl-(2,6-difluoro-phenoxy)-methyl ketone], a pancaspase inhibitor, was purchased from Sigma-Aldrich. Q-VD-OPh and Z-LEVD were dissolved in dimethyl-sulfoxide (DMSO) (Sigma-Aldrich) and bortezomib was dissolved in 0.9% NaCl. For controls, vehicle (DMSO or NaCl) was added at the same final concentration.

### Generation of cyclin D1-expressing cell lines

RPMI 8226 cells (referred to here as 8226 cells) were purchased from DSMZ (ACC-402). LP1 cells were generously provided by R Bataille (Centre de recherche en cancérologie Nantes-Angers, Nantes, France). U266 (ACC-9) and KMS-12-PE (ACC-606), from DSMZ, were used as positive control for cyclin D1 expression in immunocytochemistry analysis. Human myeloma cell lines (HMCLs) were maintained in RPMI 1640 medium (Lonza) supplemented with 2 mM L-glutamine (Lonza), 10% fetal calf serum (FCS, PAA Laboratories) and antibiotics (Lonza).

The pEGFP-N1 plasmid was purchased from Clontech Laboratories Inc. and the p-cyclin D1-EGFP plasmid was kindly provided by D. Salomon (USCF School of Medicine, San Francisco, CA, USA). This plasmid was sequenced to check the integrity of the coding sequence, amplified and purified with the QIAGEN maxi kit (Qiagen). We electroporated (250 V, 950 μF, Gene Pulser II, Bio Rad) 10^7^ 8226 or LP1 cells with 10 μg pEGF-N1 or p-cyclin D1-EGFP plasmids in RPMI 1640 medium without FCS. After incubation for 24 h, the cells were transferred to complete medium supplemented with 500 μg/mL G418 (PAA Laboratories). They were cloned by limiting dilution methods. Clones were maintained under selective pressure and selected on the basis of their FL1-fluorescence by flow cytometry (Gallios, Beckman Coulter). Data were analyzed with the Kaluza software (Beckman Coulter).

### Cell viability measurement

Cell proliferation was determined by directly counting the cells after trypan blue staining. Cell viability was quantified with the CellTiter 96® AQueous One Solution (MTT [(3-(4,5)-dimethylthiazol-2-yl)-2,5-diphenyltetrazolium bromide] assay (Promega), according to the manufacturer’s instructions.

### Apoptosis determination by APO2.7 staining and cytometry sorting

Drug-exposed HCMLs and control cells were stained with the APO2.7-PE-conjugated antibody (Ab), as described by the manufacturer (Beckman Coulter), and analyzed by flow cytometry. We analyzed at least 10^4^ cells for each set of culture conditions, and each experiment was carried out three times.

### Indirect immunofluorescence and confocal microscopy analysis

Cells (2 × 10^5^ per spot) were cytopsun on Superfrost slides at 500 g for 3 min, then fixed in 4% paraformaldehyde for 15 min and permeabilized with 0.5% Triton X100 for 5 min. Slides were processed as previously described [10], with an anti-cyclin D1 (sc-718, Santa Cruz Biotech., Santa Cruz CA) as primary Ab and AlexaFluor 633-labeled anti-rabbit IgG (Molecular Probes) as secondary Ab. Nuclei were counterstained with 4′,6-diamidino-2-phenylindole, dihydrochloride (DAPI). Slides were analyzed with a Fluoview FV1000 confocal microscope and Fluoview Viewer software (Olympus).

### Western blotting

Whole-cell lysates were obtained with M-PER® Mammalian Protein Extraction Reagent (Thermo Scientific, Rockford, Illinois, USA), and western blotting was carried out as described elsewhere [18]. We used primary Abs against poly(ADP-ribosyl)transferase (or PARP, #9542), caspase 9 (#9508), XBP1 (#7160), eIF2α (#9722), phospho(p)-eIF2α (#9721), and BiP (#3183) from Cell Signaling Tech. (Danvers, MA). The Abs against MCL1 (S-19); caspase 3 (H-277), caspase 8 (H-134), cyclin D1 (M-20), cyclin D2 (M-20), XBP1 (M-186), and β-actin (C4) were obtained from Santa Cruz Biotech. The Ab against GAPDH (clone 6C5) was obtained from Life Technologies; that against caspase 4 (M029-3) was obtained from MBL and the Ab against BCL2 (M0887) was obtained from Dako. The secondary Abs used were goat anti-rabbit or anti-mouse peroxidase-conjugated IgGs (Abcam).

### RNA extraction, quantification, amplification and real-time polymerase chain reaction

RNA was extracted with the RNAeasy® Mini kit (Qiagen), according to the manufacturer’s instructions, and quantified with a Smartspec™ 3000 spectrometer (Bio-Rad). We generated cDNAs from 2 μg of RNA with M-MuLV-reverse transcriptase, according to the manufacturer’s instructions (Invitrogen, Life Technologies). The cDNA was then subjected to real-time PCR with the SYBR Green system (Applied Biosystems, Life Technologies) and primers for *RPLP0*, *GAPDH*, *CXCR3*, *BTBD3*, *MCL1*, *BCL2L1*, *RND3* and *CXCL10* (Additional file 1). *BiP*, *GRP94* and *CHOP* cDNAs were amplified as previously described [19]. We used the StepOnePlus real-time PCR System (Applied Biosystems). Data were analyzed with Step One software V2.2.2 (Applied Biosystems). Gene expression was quantified by the ΔΔ*Ct* method, with *RPLP0* used as the internal standard. The fold change in expression (Fc) was calculated as 2^-ΔΔ*Ct*^.

### Whole-genome expression analysis

Total RNA was purified from four independent cultures of 8226 GFP (Cl1), RPMI 8226 D1-GFP (Cl2), LP1 GFP (Cl4) and LP1 D1-GFP (Cl3) cells, with the RNAeasy® Mini kit (Qiagen). RNA was quantified with a Nanodrop ND-1000 spectrophotometer (Thermo Fisher Scientific Inc.). RNA integrity was assessed with a Bioanalyzer 2100 machine and the RNA6000 Nano kit (Agilent Technologies Inc.). All samples had a RNA integrity number of at least 7.00. No signs of DNA contamination were detected. We used 400 ng of total RNA per sample. We used the Illumina Total Prep RNA Amplification Kit (Ambion, Life Technologies) to generate biotinylated, amplified cRNA, according to the manufacturer’s recommendations. Hybridization on Illumina HumanHT-12 v4 Expression BeadChips, and the staining and detection of cRNAs with the I-Scan system were performed according to the manufacturer’s recommendations (Illumina Inc., San Diego, USA). The HumanHT-12 v4 Expression BeadChip assesses a total of 47,231 marker probes, 28,688 of which are NM coding transcripts; and 11,121 are XM coding transcripts (RefSeq Content, Build 33.1, Release 5). It also contains 3,461 experimentally confirmed mRNA sequences that have been aligned with EST clusters. GenomeStudio 2011.1 software (Illumina Inc.) and its Gene Expression Analysis Module (version 1.9.0) were used for signal extraction and quantile normalization. Processed probe data were then filtered according to the following criteria: minimal signal intensity fold-change of 1.50 across all samples; minimal absolute change in probe signal intensity of 150 across all samples, based on the variance of gene expression, with a 10% threshold. The filtered data were then log-transformed and exported to appropriate software for secondary analyses. Omics Explorer 2.2 software (Qlucore, Lund, Sweden [20]) was used for hierarchical clustering and analyses of differential expression. An adjusted *p*-value (*q*-value) of 0.01 was considered as significant in the differential expression analyses. The primary probe data were subjected to quantile normalization. Genes were considered to be differentially expressed if the absolute fold-change (Fc) in mean expression values between D1-GFP-expressing cells and GFP-expressing cells was at least 1.5.

### Statistical analysis

Student’s *t*-test was used to determine the significance of differences between two experimental groups. Data were analyzed in two-tailed tests, with *p* < 0.05 (*) considered significant and *p* < 0.01 (**) considered highly significant.

## Results

### Cyclin D1 is expressed and functional in 8226- and LP1-derived clones

**Figure 1:**
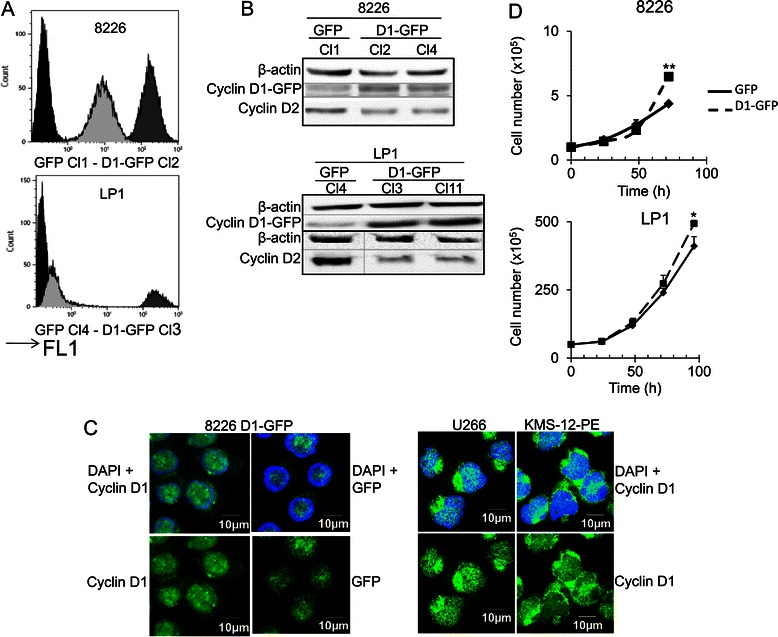
Cyclin D1 is expressed in 8226- and LP1-derived clones. **A**. The GFP fluorescence (FL1) of parental cells and the clones derived from them were analyzed by flow cytometry. Representative profiles are shown from the selected clones: in black, parental cells; light gray: cyclin D1-GFP clone; dark gray: GFP clone. **B**. Whole-cell proteins extracts were obtained from GFP- and cyclin D1-GFP-expressing cells, and subjected to SDS-PAGE and immunoblotting with the indicated Abs. An Ab directed against β-actin was used as loading control. The 8826 D1-GFP Cl2 and LP1 D1-GFP Cl3 clones were used for the experiments shown in parts A and D of this figure and for subsequent investigations. **C**. 8226 cells expressing exogenous cyclin D1 or U266 and KMS-12-PE cells expressing endogenous cyclin D1 were stained with anti-cyclin D1 Ab then with AlexaFluor 633-conjugated anti-mouse IgG (in green) and couterstained with DAPI (in blue). Slides were analyzed by confocal microscopy (Fluoview microscope). **D**. Clones presented in A were used to seed complete medium at a density of 2 × 10^5^ cells/ml, and their growth was evaluated daily, over a three- or four-day period, by direct counting after trypan blue staining. The experiment was carried out three times in triplicate. The mean number of living cells is shown, together with the SD. *p < 0.05; **p < 0.01 in Student’s *t*-test.

We investigated the role of cyclin D1, in two HMCLs (8226 and LP1 cells), from molecular groups of MM with a poor prognosis [17,21], in which cyclin D1 protein was barely detected. Cells were transfected by electroporation with plasmids encoding GFP or D1-GFP and were selected for neomycin resistance. Clones for each HMCL were obtained by limiting dilution cloning and their protein levels were quantified by flow cytometry (Additional file 2). The GFP-expressing clones obtained from the two parental cell lines were highly fluorescent, suggesting that the electroporation conditions were optimal (Figure 1A). However, D1-GFP protein levels were only moderate in 8226 cells and low in LP1 cells. The presence of the D1-GFP fusion protein in these clones was confirmed by immunoblotting with an anti-cyclin D1 Ab (Figure 1B). Consistent with previous observations [16], an upregulation of cyclin D1 correlated with a downregulation of cyclin D2 (Figure 1B). According to other reports [22-24], high levels of cyclin D1 are deleterious for MM cell survival, and only clones with moderate or low cyclin D1 levels survive. Like cyclin D1, the GFP-cyclin D1 fusion protein was present in both the nucleus and cytoplasmic compartments (Figure 1C). We then compared the proliferation properties of D1-GFP- and GFP-expressing clones. As expected, clones expressing cyclin D1 displayed high levels of cell proliferation (Figure 1D). Our data demonstrated that, despite its low level of expression, cyclin D1 affects HMCL behavior.

### Cyclin D1 expression alters various cellular processes including cell death/apoptosis

**Figure 2:**
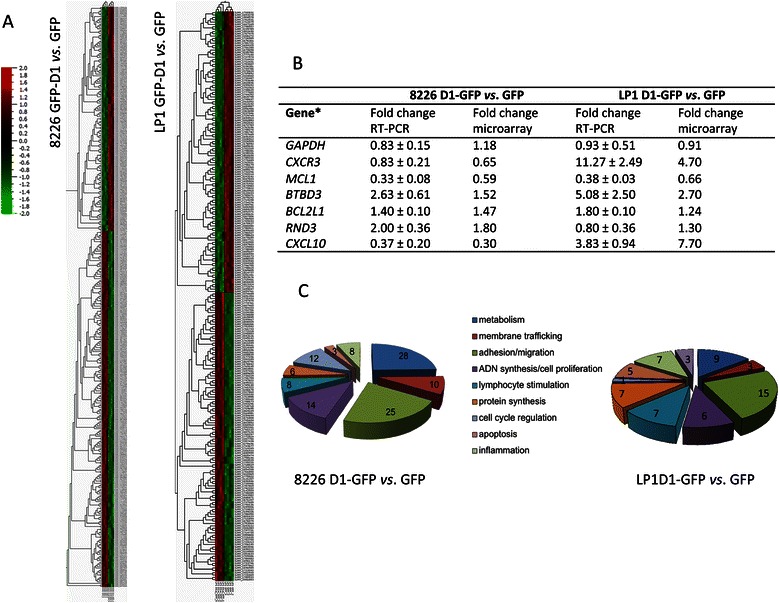
Cyclin D1 expression in HCMLs alters various molecular functions. **A**. The whole-genome expression patterns for 8226 D1-GFP *vs*. 8226 GFP (left) and LP1 D1-GFP *vs*. LP1 GFP (right) are shown as heat maps. Each column represents a sample; each row represents a marker (gene transcript). The log2 relative gene expression scale is shown at the bottom left. The differentially expressed genes (Fc threshold ≥ 1.5, q = 0.01) were classified as cyclin D1-activated genes (in red) or cyclin D1-inhibited genes (in green). **B**. Real-time quantitative RT-PCR for the validation of microarray data. The sequences of the primers used for RT-PCR are indicated in the Additional file 1. For each sample, the mean Ct value for the internal standard (*RPLP0*) was subtracted from the mean Ct value for each gene, to obtain the ΔΔCt. The formula N = 2^-ΔΔCt^ was used to calculate the Fc in expression. The experiments were carried out three times; mean ± SD values are indicated in the table. For each gene, the Fc calculated from microarray data is indicated in the column on the right. **C**. Genes for which expression levels were altered (Fc > 2) by cyclin D1 expression were ordered by function-related groups, for both cell lines. The number of genes with altered expression levels corresponding to each cell function is indicated on the graph.

We selected one clone from each 8226 and LP1 series for gene expression analysis. For each clone (8226 GFP Cl1, 8226 D1-GFP Cl2, LP1 GFP Cl4, LP1 D1-GFP Cl3), total RNA was purified from four independent cultures and used for whole-genome transcription profiling. The microarray data and annotations have been deposited in the NCBI/NIH Gene Expression Omnibus (GEO) under the accession number [GSE59673]. As illustrated by the heatmaps obtained, cyclin D1 had a significant impact on gene transcription in both cell lines (Figure 2A). Real-time RT-PCR was performed to validate the microarray results. Microarray and RT-PCR data were particularly well correlated for seven genes differentially regulated between the GFP-D1 series and GFP series (regardless of whether expression was upregulated or downregulated in the presence of cyclin D1; Figure 2B). The differentially expressed genes were then subjected to hierarchical clustering on the basis of their biological functions, with DAVID Bioinformatics Resources 6.7 [25]. Many genes involved in DNA synthesis/cell proliferation, cell cycle regulation, or adhesion/migration were characterized (Figure 2C); cyclin D1 has well known roles in these functions [4-13]. Moreover, we found, in both cell models, changes in transcription levels for genes involved in metabolism, protein synthesis, membrane trafficking, lymphocyte stimulation, inflammation and cell death/apoptosis, functions not previously associated with cyclin D1. We then carried out a functional enrichment analysis for the differentially expressed genes with DAVID, focusing on genes displaying a Fc in expression > 2. Four (for 8226 cells) and five (for LP1 cells) gene ontology clusters were identified including two clusters common to both series: inflammatory response and cell death/apoptosis (Table 1). We then focused on the role of cyclin D1 in regulating apoptosis.

**Table 1:** **Enrichment of differentially expressed genes in Gene Ontology terms**

Cluster	Gene ontology term	Enrichment score	No. of genes	Total genes	% of genes
**Clusters for RPMI D1-GFP** ***vs*** **. GFP**					
1	Inflammatory response	5.03	27	108	25.00
2	Extracellular matrix binding	3.45	9	116	7.76
3	Extracellular space	2.66	29	129	22.48
4	Cell death/apoptosis	1.93	13	108	12.04
**Clusters for LP1 D1-GFP** ***vs*** **. GFP**					
1	Antigen processing	5.58	7	48	14.58
2	Inflammatory response	2.05	5	48	10.42
3	Bone development	0.96	3	48	6.25
4	Developmental process	0.88	17	48	35.42
5	Cell death/apoptosis	0.72	4	48	8.33

### Cyclin D1 sensitizes HMCLs to bortezomib by increasing caspase-dependent apoptosis driven by the intrinsic pathway

**Figure 3:**
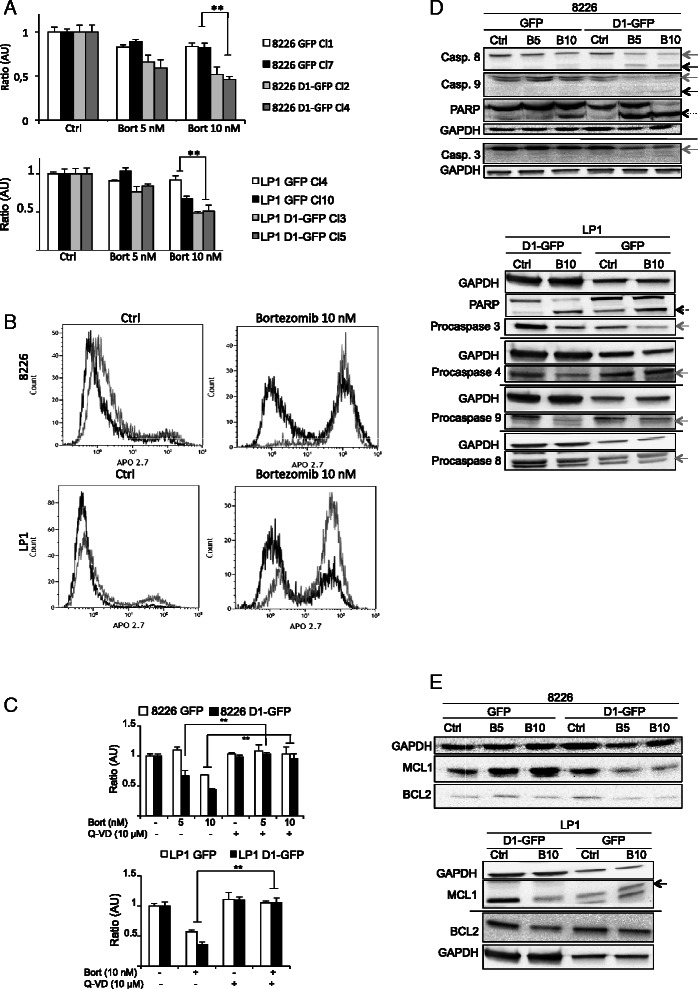
Cyclin D1 expression amplifies a caspase-dependent apoptosis. **A**. 8226- and LP1-derived clones were treated for 24 h with vehicle (Ctrl) or bortezomib (Bort, 5 or 10 nM). Cell viability was quantified in MTT assays. The absorbance (OD at 490 nm) of each clone treated with the drug is expressed relative to that of the corresponding clone treated with vehicle (defined as 1 in arbitrary unit, AU). For each set of culture conditions, the mean of triplicate ratios is indicated on the graph, together with the SD. ** *p* < 0.01. **B**. GFP- (in black) or D1-GFP-expressing clones (in gray) were treated with vehicle (Ctrl) or 10 nM bortezomib. After 24 h, the cells were stained with anti-APO2.7 Ab and analyzed by flow cytometry. At least 2 × 10^4^ events were gated for each clone and set of culture conditions. Representative profiles of APO2.7-stained cells are shown. The quantification of APO2.7-positive cells in several independent experiments is reported in the Additional file 3. **C**. Cells were treated with the pancaspase inhibitor the Q-VD-OPh (or vehicle) for 1 h before treatment with bortezomib (or vehicle) at the concentrations indicated for an additional 24 h. Cell viability was measured as described in A. Each experiment was carried out two or three times, in triplicate. ** *p* < 0.01. **D**. Cells were treated as described above for 24 h. Whole-cell protein extracts were obtained and separated by SDS-PAGE. Proteins were blotted and analyzed with the Abs indicated. The GAPDH Ab was used as a loading control. The proforms of caspase (Casp.) are indicated by gray arrows; the cleaved, activated forms of caspase are indicated by black arrows. Cleaved PARP is indicated by a dotted arrow. **E**. The cells were treated as described above for 24 h. Whole-cell protein extracts were prepared and separated by SDS-PAGE. Proteins were blotted and analyzed with the Abs indicated. The GAPDH Ab was used as a loading control. MCL1 protein is indicated by an arrow.

Cyclin D1 has been reported to play a direct role in the regulation of apoptosis and, thus, in the resistance/sensitization of tumor B cells to anticancer drugs. However, depending on the differentiation state of cells of the B-cell lineage, cyclin D1 may have anti-apoptotic effects [26,27] or may increase chemosensitivity [28]. We investigated the effects of cyclin D1 on the apoptotic response, by treating clones expressing GFP or D1-GFP with bortezomib, a proteasome inhibitor widely used to treat MM and B-cell hemopathies [29]. Cell viability was assessed in a MTT assay. We first checked that the GFP-expressing cells and the corresponding parental cells had similar sensitivity (data not shown). The expression of cyclin D1 was associated with significantly lower levels of cell viability after bortezomib-treatment in both HMCLs (Figure 3A). Thus, cyclin D1 expression amplified the response of HMCLs to bortezomib.

One clone from each series (8226 D1-GFP Cl1, 8226 D1-GFP Cl2, LP1 GFP Cl4, LP1 D1-GFP Cl3) was studied further. These clones were stained with APO2.7-PE Ab and sorted by FACS for the quantification of apoptosis. The percentage of apoptotic cells (APO2.7-positive) after bortezomib-treatment was higher for 8226- and LP1-D1-GFP cells than for GFP-expressing clones (Figure 3B and Additional file 3). Treatment of the 8226 and LP1 clones with the Q-VD-OPh pancaspase inhibitor before bortezomib treatment abolished the difference in response between GFP- and D1-GFP-expressing clones (Figure 3C). These findings confirmed that cyclin D1 potentiated caspase-dependent apoptosis in HMCLs.

Caspase-dependent apoptosis is a well defined type of programmed cell death controlled by an extrinsic pathway after death receptor activation and/or by an intrinsic pathway regulated by proteins of the B-cell lymphoma 2 (BCL2) family. Bortezomib induces caspase 8- and 9-dependent apoptosis [30]. The treatment of 8226 D1-GFP cells with bortezomib was sufficient to induce the cleavage of procaspases 8, 9 and 3 (illustrated by the disappearance of procaspase forms and the appearance of active shorter forms) and, in turn, the cleavage of PARP (Figure 3D). No such cleavage events were observed in 8226 GFP cells. Similar results were obtained for bortezomib-treated LP1 cells (Figure 3D). Apoptosis was triggered in both GFP- and D1-GFP-expressing cells, but cyclin D1 amplified the response.

The anti-apoptotic BCL2 and myeloid leukemia cell differentiation 1 (MCL1) proteins are key regulators of apoptosis in MM. MCL1, in particular, is essential for the survival of plasma cells and its degradation is required for MM cell death [31]. Bortezomib treatment decreased BCL2 and MCL1 levels in a dose-dependent manner in both D1-GFP- and GFP-expressing clones, but this effect was stronger in cyclin D1-expressing cells (Figure 3E). We concluded that the higher chemosensitivity of D1-GFP-expressing clones resulted from early procaspase activation and triggering of the intrinsic apoptotic pathway. In MM cells, apoptosis is also controlled by the UPR pathway [32]. We therefore investigated the possible effects of cyclin D1 on the signaling associated with this pathway.

### Cyclin D1 deregulates the pro- and anti-apoptotic balance of the UPR after drug treatment

ER stress upregulates the UPR *via* three distinct ER-resident proteins: activating transcription factor 6 (ATF6), protein kinase R (PKR)-like endoplasmic reticulum kinase (PERK) and inositol-requiring enzyme 1 (IRE1). These proteins activate different transcriptional and translational programs [33]. The accumulation of unfolded proteins in the ER lumen leads to dissociation of the chaperones BiP (immunoglobulin binding protein or the 78 kDa glucose-regulated protein) and, to some extent, Grp94 (94 kDa glucose-regulated protein or HSP90B1), from IRE1, PERK and ATF6, leading to the activation of these ER stress sensors. In myeloma cells, the interplay between these proteins determines the outcome of ER stress: survival or death [33].

**Figure 4:**
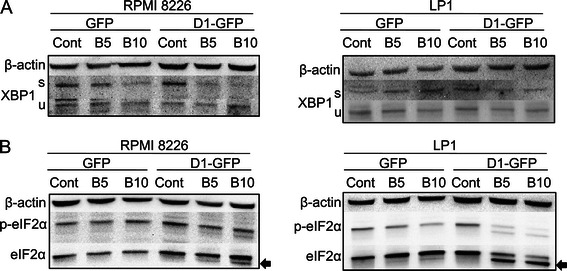
Cyclin D1 expression impairs the IRE1 and PERKS pro-apoptotic axes of the UPR after bortezomib treatment. 8226- and LP1-derived clones were treated with vehicle (Cont) or treated with 5 or 10 nM bortezomib for 24 h. The cells were then harvested, and whole-cell proteins were prepared and analyzed by SDS-PAGE and in Immunoblotting with anti-XBP1 **(A)** or EIF2α **(B)** Abs. The anti-β-actin Ab was used as a control for charge and transfer. The experiments were carried out two times; one is presented. The spliced and active form of XBP1 is denoted “s”, the unspliced form is denoted “u”; the cleaved form of eIF2α is indicated by an arrow.

Activated IRE1 acts as an endoribonuclease, cleaving X box-binding protein (XBP1) mRNA. The spliced form of XBP1, XBP1s, is a transcription factor essential for myeloma cells survival [34]. The blockade of IRE1 endonuclease activity is associated with cell death [35]. GFP- and D1-GFP-expressing clones from the two HMCLs were treated with various concentrations of bortezomib, which induces the UPR [36]. Whole-cell proteins extracts were then obtained and analyzed by western blotting. Consistent with published data for MM patients [35], XBP1s was found to be present in the two HMCLs, whereas its levels were decreased by the ER stress imposed by bortezomib treatment (Figure 4A). In both HCMLs, cyclin D1 expression accelerated the disappearance of XBP1s. We concluded that cyclin D1 blocks the survival-promoting XBP1-mediated UPR pathway.

The PERK/eukaryotic initiation factor 2 α (eIF2α)/ATF4 axis may protect cells or trigger cell death [32]. PERK activation leads to the phosphorylation of eIF2α, the activation of ATF4, and the transcription of genes encoding ER chaperones and other targets [33]. The phosphorylated form of eIF2α (p-eIF2α) was present in all HCMLs (Figure 4B), consistent with constitutive activation of the PERK/eIF2α signaling pathway. Following bortezomib treatment, eIF2α was cleaved in a dose-dependent manner in cyclin D1-GFP-expressing cells, but not in GFP-expressing cells, suggesting that it was targeted by proteases (possibly caspases). Overall, cyclin D1 expression impaired UPR-mediated survival programs.

### CHOP is targeted by cyclin D1 to signal cell death

**Figure 5:**
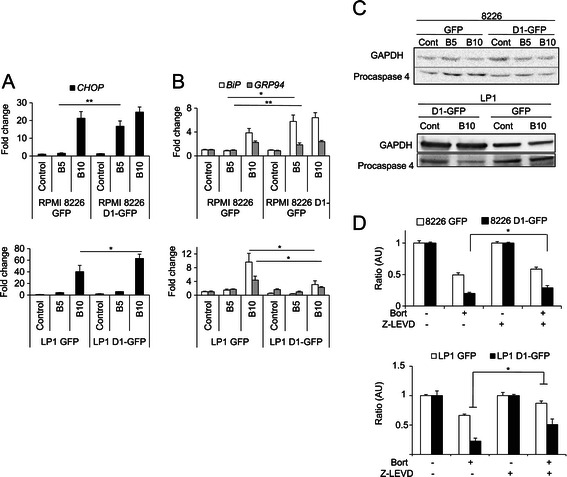
Cyclin D1 activates the UPR pro-apoptotic pathway after bortezomib treatment. Cells were treated with bortezomib (B, 5 or 10 nM) or vehicle (control) for 24 h. The expression of the *CHOP* (**A**), *BiP* and *GRP94* (**B**) genes was determined by RT quantitative-PCR. For each series, the mean of triplicate samples is presented, together with the SD. All experiments were carried out three times. *p < 0.05, **p < 0.01. **C**. Cells were treated as described above. Proteins were blotted on membranes and probed with an anti-caspase 4 Ab. The anti-GAPDH Ab was used as a loading control. **D**. Cells were treated with a caspase 4 inhibitor (Z-LEVD, 1 μM) for 1 h, and then with vehicle or bortezomib (7.5 nM for 8226 or 10 nM LP1, respectively) for another 24 h. Cell viability was estimated in MTT assays. *p < 0.05.

Each of the three sensors of the UPR simultaneously activates pro- and anti-apoptotic signals [37]. For example, PERK release and oligomerization lead to eIF2α phosphorylation, resulting in the downregulation of translation; a prosurvival mechanism. However, activation of the PERK/eIF2α/ATF4 axis leads to transcription of the downstream target gene *CHOP* and, in turn, to the transcription of effectors driving mitochondria-dependent and -independent apoptosis [38]. CCAAT/enhancer binding protein homologous protein or CHOP (also known as GADD153) is the executioner molecule in ER-mediated apoptosis. There is crosstalk between the PERK and ATF6 sensor systems and the signaling pathways for both these systems converge in the activation of *CHOP* transcription [33]. Total RNA was purified from HCMLs after bortezomib treatment and analyzed by RT-quantitative PCR. The Fc values obtained indicated that cyclin D1 expression significantly increased the expression of *CHOP* (Figure 5A). These data suggest that a specific CHOP-mediated ER stress response was initiated by the presence of cyclin D1 in bortezomib-treated cells. By contrast, the expression of *BiP* and *GRP94*, downstream from PERK activation, was affected in different ways in different HCMLs (Figure 5B). Caspase 4 is the recognized ER-specific executioner caspase [37]. The activating cleavage of caspase 4 was enhanced in cyclin D1-expressing cells (Figure 5C). Moreover, prior treatment of the cells with the caspase 4 inhibitor Z-LEVD partially restored cell viability in D1-GFP-expressing clones (Figure 5D). We concluded that a cell death program induced by the UPR and mediated by the transcription factor CHOP was amplified by cyclin D1 in bortezomib-treated HCMLs.

Our data demonstrate that cyclin D1 expression favors a pro-apoptotic UPR pathway, increasing the sensitization of HMCLs to bortezomib.

## Discussion

MM is relatively homogeneous in terms of its histological, clinical and biologic properties, but it is characterized by genetic and phenotypic complexities with implications for treatments. Gene expression profiling assays have confirmed that MM patients from the CD-1/2 group (MM expressing cyclin D1) have a better prognosis than those from the MS and MF groups (MM expressing cyclin D2) [17]. We investigated the molecular basis of this behavior, by expressing cyclin D1 in HMCLs belonging to groups of MM patients with a poor prognosis. We studied the 8226 and LP1 cell lines because they produce little or no cyclin D1. The constitutive expression of cyclin D1 is cytotoxic in B cells [24]. We therefore obtained cell clones producing only moderate to low levels of cyclin D1. Nevertheless, cyclin D1 expression was found to be associated with higher levels of cell proliferation and the downregulation of cyclin D2, as previously reported [16,28]. These observations are consistent with previous reports that MM of the CD-1/2 group respond rapidly to treatment, and that their response rates are high. However, the duration of complete responses has been shown to be shorter for this group of tumors than for other types of MM [39], possibly due to the higher rates of proliferation of cyclin D1-expressing tumor cells.

Comparison of the transcriptional profiles of 8226 and LP1 with and without cyclin D1 expression showed that many genes were up- or down-regulated in the presence of cyclin D1. However, only a small number of genes were coordinately regulated by cyclin D1 in both cell types (Additional file 4). We then focused on the cell functions controlled by cyclin D1. The genes differentially expressed in both cell types were enriched in genes associated with apoptosis/cell death and inflammation. We then investigated the potential mechanism by which cyclin D1 might modulate the apoptotic response.

Cyclin D1 expression significantly increased the apoptosis of 8226 and LP1 cells after a bortezomib treatment. This finding is consistent with those obtained by Kuroda *et al.* for RPMI 8226 cells [28] and indicates that the expression of cyclin D1 amplifies the response when the apoptotic machinery is activated.

We found that cyclin D1 affected at least two of the three branches of UPR and enhanced an ER stress-induced apoptosis, regardless of genetic background of the HMCL. Plasma cells are terminally differentiated B lymphocytes that secrete immunoglobulins and have a high demand for protein synthesis and exocytosis [40]. The survival of MM cells is highly dependent on the UPR, and inefficient or prolonged UPR activation generates ER stress that can signal apoptosis [41,42]. In MM cells, the three arms of the ER stress program are activated, but the CHOP response is limited. Moreover, MM cells can increase the ER stress response further, with ATF4 and ATF6 coordinately inducing *CHOP* transcription. This causes a shift in the ER stress from a protective to a destructive mechanism. Bortezomib, a potent antimyeloma compound, takes advantage of this property and upregulates PERK activity [36]. In the cell models we developed, cyclin D1 expression disrupted the UPR balance, favoring death and amplifying the effects of bortezomib on the PERK/CHOP axis.

The mechanism by which cyclin D1 modifies the UPR has not yet been determined. ATP, Ca^2+^ and an oxidizing environment are required for correct protein folding [43]. We previously reported that cyclin D1 inhibits mitochondrial activity in B-cell lymphoma, by competing with hexokinase 2 for binding to the voltage-dependent anion channel [10]. Cyclin D1 binding modifies the flux of ATP/ADP/Pi metabolites, with a potentially major impact on protein synthesis and folding. Further experiments are required to confirm this hypothesis in our cell models.

Pro- and antiapoptotic members of the BCL2 family are crucial regulators of apoptosis in plasma cells. Several studies have demonstrated that BCL2 and MCL1 are essential for the survival of plasma cell survival [44,45]. Consistent with these findings, BCL2 and MCL1 levels rapidly decrease in cyclin D1-expressing cells (*i.e.* in cells programmed for death). The MCL1 and BCL2 proteins regulate the apoptotic pathway mediated by mitochondria, but they are also involved in ER stress-mediated apoptosis. Indeed, chemical treatments or changes in the microenvironment lead to severe ER stress, initiating apoptosis. This process is mediated by the induction of CHOP, which eliminates BCL2, thereby counteracting the anti-apoptotic function of this protein [37]. It has recently been shown, in MM cells, that the UPR and, more specifically, the PERK-eIF2α-ATF4 branch of this pathway, regulates MCL1 levels [46,47]. Thus, both the apoptotic and UPR pathways are involved in the degradation of BCL2 and MCL1. We found that caspase activation was correlated with the decrease in BCL2 and MCL1 levels after bortezomib-treatment. Bortezomib inhibits the proteasome, so the degradation of BCL2 and MCL1 may be essentially due to caspase-mediated cleavage, as previously suggested [48-50], rather than degradation by the proteasome [51]. This would be consistent with a master role for caspases in the downregulation of BCL2 and MCL1 after bortezomib treatment.

## Conclusion

We report here that cyclin D1-expressing MM cells are particularly sensitive to ER-stress and the UPR. Our results suggest that drugs targeting UPR effectors may act in synergy with bortezomib, sensitizing MM cells with a poor prognosis to bortezomib and improving the clinical response of patients.

## Additional files

Additional file 1:
**Sequences of the primers used in the study.**

Additional file 2:
**Flow cytometry analysis of** **GFP-** **and cyclin** **D1-GFP-expressing** **clones.**

Additional file 3:
**Quantification of APO2.7-positive cells after vehicle or drug treatments.**

Additional file 4:
**Genes altered by cyclin D1 expression in RPMI 8226 and LP1 cells.**

## Abbreviations

Ab: Antibody
ATF4/6: Activating transcription factor 4/6
BCL2: B-cell lymphoma 2
BiP: Immunoglobulin binding protein or 78 kDa glucose-regulated protein or Grp78
CHOP: CCAAT/enhancer binding homologous protein (or GADD153)
DAPI: 4′,6-diamidino-2-phenylindole, dihydrochloride
DMSO: Dimethylsulfoxide
eIF2α: eukaryotic initiation factor 2α
ER: Endoplasmic reticulum
GFP: Green fluorescent protein
Fc: Fold change
Grp94: 94 kDa glucose-regulated protein or heat shock protein 90 kDa
HMCL: Human myeloma cell line
IRE1: Inositol-requiring enzyme 1
MCL1: Myeloid cell leukemia 1
MM: Multiple myeloma
PERK: PKR-like endoplasmic reticulum kinase
PKR: Protein kinase R
UPR: Unfolded protein response
