# Supplementary material for: Cyclin D1 sensitizes myeloma cells to endoplasmic reticulum stress-mediated apoptosis by activating the unfolded protein response pathway
Source: BMC Cancer. 2015 Apr 11;15:262. doi: 10.1186/s12885-015-1240-y (PMC4399746; doi:10.1186/s12885-015-1240-y)
Supplement: Additional file 1: — Sequences of the primers used in the study. [file 12885_2015_1240_MOESM1_ESM.docx]

**Additional File 1**. Sequences of the primers used in the study

| Gene | Forward 5' -> 3' | Reverse 5'-> 3' |
| --- | --- | --- |
| *RPLP0* | CCA GGC GTC CTC GTG GAA GTG | TTC CCG CGA AGG GAC ATG CG |
| *GAPDH* | CTG ACT TCA ACA GCC ACA CC | CCC TGT TGC TGT AGC CAA AT |
| *CXCR3* | CTT GAG GTG AGT GAC CAC CAA GTG | GCA GGA AGG CCC GGT CGA AG |
| *BTBD3* | CTC ACG CAG CTC CAG VVV ATC | TGC TGC TTT TCT CAG CCA GGC AAG |
| *MCL1* | TCG GCC CGG CGA GAG ATA GG | TCC GGG AGT CTG GCG TGA GG |
| *BCL2L1* | GGG AGG CAG GCC ACG AGT TT | CAC AGT GCC CCG CCG AAG GA |
| *RND3* | GGG ATG CGA GTA CCT CCA AG | TGC TAA GGT CAC AGT GCC AG |
| *CXCL10* | TGA AAT TAT TCC TGC AAG CCA A | GTA GCA ATG ATC TCA ACA CGT GG |
