# Supplementary material for: Cyclin D1 sensitizes myeloma cells to endoplasmic reticulum stress-mediated apoptosis by activating the unfolded protein response pathway
Source: BMC Cancer. 2015 Apr 11;15:262. doi: 10.1186/s12885-015-1240-y (PMC4399746; doi:10.1186/s12885-015-1240-y)
Supplement: Additional file 2: — Flow cytometry analysis of GFP- and cyclin D1-GFP-expressing clones. [file 12885_2015_1240_MOESM2_ESM.docx]

**Additional File 2**. Flow cytometry analysis of GFP- and cyclin D1-GFP-expressing clones

|  |  | MFI* | |  |
| --- | --- | --- | --- | --- |
| Cell line |  | Parental | Clone Clone | Ratio** |
| RPMI 8226 | **GFP Cl1** | **0.26** | **89** | **342.3** |
|  | GFP Cl7 | 0.26 | 163 | 626.9 |
|  | GFP Cl9 | 0.26 | 195 | 750.0 |
|  | **D1-GFP Cl2** | **0.26** | **11.4** | 43.8 |
|  | D1-GFP Cl4 | 0.26 | 7.8 | 30.0 |
|  | D1-GFP Cl6 | 0.26 | 7.6 | 29.2 |
|  | D1-GFP Cl9 | 0.26 | 5.7 | 21.9 |
|  | D1-GFP Cl10 | 0.26 | 6.1 | 23.5 |
| LP1 | GFP Cl1 | 0.19 | 251 | 1321.1 |
|  | GFP Cl2 | 0.19 | 244 | 1284.2 |
|  | **GFP Cl4** | **0.19** | **269** | **1415.8** |
|  | GFP Cl7 | 0.19 | 155 | 815.8 |
|  | GFP Cl8 | 0.19 | 170 | 894.7 |
|  | GFP Cl9 | 0.19 | 131 | 689.5 |
|  | GFP Cl10 | 0.19 | 316 | 1663.2 |
|  | D1-GFP Cl1 | 0.19 | 0.31 | 1.6 |
|  | D1-GFP Cl2 | 0.19 | 0.26 | 1.4 |
|  | **D1-GFP Cl3** | **0.19** | **0.57** | **3.0** |
|  | D1-GFP Cl4 | 0.19 | 0.31 | 1.6 |
|  | D1-GFP Cl5 | 0.19 | 0.40 | 2.1 |
|  | D1-GFP Cl6 | 0.19 | 0.31 | 1.6 |
|  | D1-GFP Cl9 | 0.19 | 0.67 | 3.5 |
|  | D1-GFP Cl11 | 0.19 | 0.51 | 2.7 |
|  | D1-GFP Cl12 | 0.19 | 0.29 | 1.5 |
| L363 | **GFP Cl2** | **0.18** | **168** | **933.3** |
|  | GFP Cl3 | 0.18 | 211 | 1172.2 |
|  | **GFP Cl4** | **0.18** | **204** | **1133.3** |
|  | GFP Cl6 | 0.18 | 186 | 1033.3 |
|  | GFP Cl7 | 0.18 | 184 | 1022.2 |
|  | **D1-GFP Cl2** | **0.18** | **0.43** | **2.4** |
|  | **D1-GFP Cl3** | **0.18** | **0.40** | **2.2** |
|  | D1-GFP Cl4 | 0.18 | 0.20 | 1.1 |
|  | D1-GFP Cl5 | 0.18 | 0.44 | 2.4 |
|  | D1-GFP Cl6 | 0.18 | 0.20 | 1.1 |
|  | D1-GFP Cl7 | 0.18 | 0.17 | 0.9 |
|  | D1-GFP Cl8 | 0.18 | 0.21 | 1.2 |
|  | D1-GFP Cl9 | 0.18 | 0.25 | 1.4 |
|  | D1-GFP Cl10 | 0.18 | 0.25 | 1.4 |

8226 cell line harbors the t(14;16) activating the c-*MAF* gene; LP1 cell line has the t(4;14) activating the *MMSET*/*FGFR3* oncogene and L363 cell line has the t(20;22) activating the *MAFB* gene. Those cell lines belong to MF, MS and CD2 MM molecular groups, respectively, associated with a bad prognosis according to [17]. Cells were transfected with expression plasmids coding for GFP or D1-GFP proteins, selected by antibiotic resistance and analyzed by flow cytometry. *MFI, mean fluorescence intensity values. **, ratio MFI clone/MFI parental. By convention, positivity is defined by a ratio > 2. The clones noted in bold were further selected and studied in details.
