# Supplementary material for: Cyclin D1 sensitizes myeloma cells to endoplasmic reticulum stress-mediated apoptosis by activating the unfolded protein response pathway
Source: BMC Cancer. 2015 Apr 11;15:262. doi: 10.1186/s12885-015-1240-y (PMC4399746; doi:10.1186/s12885-015-1240-y)
Supplement: Additional file 3: — Quantification of APO2.7-positive cells after vehicle or drug treatments. [file 12885_2015_1240_MOESM3_ESM.docx]

**Additional File 3. Quantification of APO2.7-positive cells after vehicle or drug treatments**

| Treatment | 8226 | | LP1 | |
| --- | --- | --- | --- | --- |
|  | GFP | D1-GFP | GFP | D1-GFP |
| Ctrl | 8.2 ± 4.8 | 10.3 ± 5.7 | 4.2 ± 2.8 | 12.4 ± 4.2 |
| 5 nM | 16.7 ± 8.7 | 37.3 ± 15.6* | 11.5 ± 4.6 | 32.4 ± 15.0 |
| 10 nM | 47.0 ± 15.7 | 72.6 ± 18.1* | 35.9 ± 8.9 | 68.3 ± 13.1** |

Cell lines were treated with the indicated concentrations of bortezomib for 24 h (or vehicle as control), stained with APO2.7-PE Ab and analyzed by flow cytometry. The percentage of APO2.7-positive cells is indicated in the table. Means and SD from three (for 8226 cells) or five (for LP1 cells) independent experiments are indicated. Significance of differences was calculated by comparing D1-GFP and GFP clones for each cell line, for each concentration. * *p*<0.05, ** *p*<0.01 with the Student’s *t* test.
