# Supplementary material for: Cyclin D1 sensitizes myeloma cells to endoplasmic reticulum stress-mediated apoptosis by activating the unfolded protein response pathway
Source: BMC Cancer. 2015 Apr 11;15:262. doi: 10.1186/s12885-015-1240-y (PMC4399746; doi:10.1186/s12885-015-1240-y)
Supplement: Additional file 4: — Genes altered by cyclin D1 expression in RPMI 8226 and LP1 cells. [file 12885_2015_1240_MOESM4_ESM.docx]

**Additional File 4**. Genes altered by cyclin D1 expression in RPMI 8226 and LP1 cells*

| PROBE_ID | DEFINITION | ENTREZ_GENE_ID | ILMN_GENE |
| --- | --- | --- | --- |
| ILMN_1785071 | Homo sapiens selenoprotein P, plasma, 1 (SEPP1), transcript variant 1, mRNA. | 6414 | SEPP1 |
| ILMN_1748697 | Homo sapiens lin-28 homolog B (C. elegans) (LIN28B), mRNA. | 389421 | LIN28B |
| ILMN_1791759 | Homo sapiens chemokine (C-X-C motif) ligand 10 (CXCL10), mRNA. | 3627 | CXCL10 |
| ILMN_1653278 | Homo sapiens mucin 20, cell surface associated (MUC20), transcript variant S, mRNA. | 200958 | MUC20 |
| ILMN_1667711 | Homo sapiens HRAS-like suppressor 3 (HRASLS3), mRNA. | 11145 | HRASLS3 |
| ILMN_1758281 | Homo sapiens calcitonin receptor-like (CALCRL), mRNA. | 10203 | CALCRL |
| ILMN_1774761 | Homo sapiens chemokine (C-C motif) receptor 2 (CCR2), transcript variant A, mRNA. | 1231 | CCR2 |
| ILMN_1712075 | Homo sapiens synemin, intermediate filament protein (SYNM), transcript variant B, mRNA. | 23336 | SYNM |
| ILMN_1691410 | Homo sapiens BMP and activin membrane-bound inhibitor homolog (Xenopus laevis) (BAMBI), mRNA. | 25805 | BAMBI |
| ILMN_2412192 | Homo sapiens complement factor H (CFH), transcript variant 2, mRNA. | 3075 | CFH |
| ILMN_3242540 | Homo sapiens CD163 molecule-like 1 (CD163L1), mRNA. | 283316 | CD163L1 |
| ILMN_1709153 | Homo sapiens proline rich 16 (PRR16), mRNA. | 51334 | PRR16 |
| ILMN_1702320 | Homo sapiens janus kinase and microtubule interacting protein 2 (JAKMIP2), mRNA. | 9832 | JAKMIP2 |

* 141 genes were altered in 8226 D1-GFP vs. GFP, 73 in LP1 D1-GFP vs. GFP. Among them 128 were specific to 8226 and 60 to LP1. Thirteen were common to both cell lines. However, only six of these genes were coordinately regulated : *CXCL10* and *CCR2* downregulated; *CFH*, *CD163L1*, *PRR1* and *JAKMIP2* upregulated.
